# Rift valley fever viral load correlates with the human inflammatory response and coagulation pathway abnormalities in humans with hemorrhagic manifestations

**DOI:** 10.1371/journal.pntd.0006460

**Published:** 2018-05-04

**Authors:** Annabelle de St. Maurice, Jessica Harmon, Luke Nyakarahuka, Stephen Balinandi, Alex Tumusiime, Jackson Kyondo, Sophia Mulei, Annemarion Namutebi, Barbara Knust, Trevor Shoemaker, Stuart T. Nichol, Anita K. McElroy, Christina F. Spiropoulou

**Affiliations:** 1 Viral Special Pathogens Branch, US CDC, Atlanta, GA, United States of America; 2 University of California Los Angeles, Division of Pediatric Infectious Disease, Los Angeles, CA, United States of America; 3 Uganda Virus Research Institute, Entebbe, Uganda; 4 Viral Special Pathogens Branch, US CDC, Entebbe, Uganda; 5 Kabale Regional Referral Hospital, Kabale, Uganda; 6 Emory University, Division of Pediatric Infectious Disease, Atlanta, GA, United States of America; 7 University of Pittsburgh, Division of Pediatric Infectious Disease, Pittsburgh, PA, United States of America; Saudi Ministry of Health, SAUDI ARABIA

## Abstract

Rift Valley fever virus is an arbovirus that affects both livestock and humans throughout Africa and in the Middle East. Despite its endemicity throughout Africa, it is a rare event to identify an infected individual during the acute phase of the disease and an even rarer event to collect serial blood samples from the affected patient. Severely affected patients can present with hemorrhagic manifestations of disease. In this study we identified three Ugandan men with RVFV disease that was accompanied by hemorrhagic manifestations. Serial blood samples from these men were analyzed for a series of biomarkers specific for various aspects of human pathophysiology including inflammation, endothelial function and coagulopathy. There were significant differences between biomarker levels in controls and cases both early during the illness and after clearance of viremia. Positive correlation of viral load with markers of inflammation (IP-10, CRP, Eotaxin, MCP-2 and Granzyme B), markers of fibrinolysis (tPA and D-dimer), and markers of endothelial function (sICAM-1) were all noted. However, and perhaps most interesting given the fact that these individuals exhibited hemorrhagic manifestations of disease, was the finding of a negative correlation between viral load and P-selectin, ADAMTS13, and fibrinogen all of which are associated with coagulation pathways occurring on the endothelial surface.

## Author summary

Rift Valley fever virus is an emerging virus of public health significance. In endemic areas up to 60% of the population are seropositive by adulthood. This implies that most of the individuals living in these endemic areas become infected with this virus at some point in their lifetime. This highly prevalent virus is poorly understood likely secondary to lack of diagnostic capacity in affected areas. In fact, the majority of cases are undocumented and spontaneously resolve. However, in some individuals the disease can be very severe and at times associated with hemorrhage or encephalitis. In this report we identify three men infected during the acute stage who also exhibited hemorrhagic manifestations. These men had elevated cytokine and chemokine levels consistent with immune activation. Our data show that biomarkers of fibrinolysis correlated positively with virus while biomarkers of coagulation correlated negatively with virus, suggesting that opposing hemostatic pathways are altered during disease, and that they correct with resolution of viremia.

## Introduction

Rift Valley Fever Virus (RVFV) was recently re-classified in the *Phenuivirdae* family [1], genus *Phlebovirus* [2]. Rift Valley Fever (RVF) outbreaks in animals can result in a large number of livestock deaths, including spontaneous abortions in pregnant livestock [3]. Transmission to humans can occur through direct contact with an animal’s infected tissue or bodily fluid or fomites. RVFV can also be transmitted via mosquito bite to both animals and humans [3]. Humans are at particularly increased risk of acquiring RVFV infection while assisting with animal birth or during animal slaughter. For these reasons, populations at high risk of RVF include herdsmen, butchers and abattoir workers [4]. Outbreaks of RVF have occurred throughout Africa and the Arabian Peninsula. Recent outbreaks have occurred in South Africa, Kenya, Sudan, Saudi Arabia, Tanzania, and Yemen [3, 5].

Humans infected with RVFV can present with varying degrees of symptomatology ranging from asymptomatic or a mild influenza-like illness to severe disease with hepatitis, retinitis or encephalitis [6]. Laboratory findings in patients with RVF can include leukopenia, thrombocytopenia, and elevated liver enzymes. About 1% of RVF cases may progress to hemorrhagic disease. The overall fatality rate is estimated to be 0.5–1%, however in the 2006–2007 outbreak in Kenya, the case fatality rate was reported to be as high as 29% [7].

It is not well understood why there is such variation in the clinical presentation of RVF. The interaction of viral proteins with the host immune response may play a role in pathogenicity. RVFV is an enveloped negative strand RNA virus consisting of three RNA segments varying in size: large (L), medium (M) and small (S). The S segment encodes the N and NSs proteins; the M segment encodes the Gn, Gc, and NSm proteins; whereas the L segment encodes the polymerase [2]. The NSs protein is a non-structural protein that interacts with host transcription factors and may down regulate the host innate immune response during infection [8, 9].

The interaction between the immune system, coagulation, and the endothelium during hemorrhagic fever virus infection has been previously studied in human subjects with Lassa, Ebola, and dengue virus infections [8, 10–17]. However, there are few studies of human immune responses during RVFV infection and none that examine the endothelium or coagulation pathway [8, 18, 19]. A study of South African patients infected during a 2010/2011 RVF outbreak found that fatal RVF cases had increased levels of the pro-inflammatory cytokines IL-8, IP-10, CXCL9, and MCP-1 as well as increased levels of the anti-inflammatory cytokine IL-10 [18]. Genetic polymorphisms in innate immune pathways, including IL-6, may also contribute to variations in severity of RVFV symptoms [19]. These data suggest that dysregulation of the immune response during RVFV infection may contribute to pathogenesis.

In March 2016, two human cases of RVF were identified in Uganda[20]; these were the first RVF cases in Uganda since 1968 [21]. A third RVF case was later identified in June 2016. These three cases provided a unique opportunity to examine the physiologic consequences of RVFV infection in the human host using blood samples collected serially as part of clinical care. We analyzed the concentration of a panel of cytokines/chemokines, as well as biomarkers of endothelial function and coagulopathy to look for associations between these pathways, host disease, and viral replication over time.

## Methods

### Ethics statement

This study used de-identified pre-existing specimens and clinical records obtained during an outbreak and was determined to be exempt by the Centers for Disease Control and Prevention Human Research Protection Office. CDC Protocol Number: 6920.

### Multiplex assays

Blood samples obtained in EDTA tubes as part of routine clinical care for RVF patients were analyzed using commercial multiplex assays according to the manufacturer’s instructions. The samples were aliquoted, frozen, and gamma irradiated on dry ice with 5x10^6^ rads. In preparation for use in the assays, samples were thawed and centrifuged to remove membrane particles. The freeze thaw process and use of whole blood in these assays were previously demonstrated using 8 human blood samples from healthy controls obtained in the US [22]. Eight healthy human samples were also used to define the normal range for each analyte in this study. These samples were obtained via a normal healthy phlebotomy program in Atlanta, GA. Sex, age and race/ethnicity of the donors were unknown. Fifty-nine analytes were assessed in ten commercially available assays from Invitrogen (Carlsbad, CA, USA), Millipore (Billerica, MD, USA), and Affymetrix (Santa Clara, CA, USA). The largest of these was a 30-plex assay for granulocyte colony stimulating factor (G-CSF), granulocyte macrophage colony stimulating factor (GM-CSF), interferon (IFN)-α, IFN-γ, interleukin (IL)-1β, IL-1RA, IL-2, IL-2R, IL-4, IL-5, IL-6, IL-7, IL-8, IL-10, IL-12 (p40/p70), IL-13, IL-15, IL-17, tumor necrosis factor (TNF)-α, Eotaxin, interferon gamma induced protein 10 (IP-10), monocyte chemoattractant protein (MCP)-1, monokine induced by gamma interferon (MIG), macrophage inflammatory protein (MIP)-1α, MIP-1β, regulated on activation normal T-cell-expressed and secreted (RANTES), endothelial growth factor (EGF), fibroblast growth factor (FGF)-basic, hepatocyte growth factor (HGF), and vascular endothelial growth factor (VEGF) that was performed using an overnight incubation (Invitrogen). Five additional assays also incubated overnight included three singleplex assays for Ferritin, ADAMTS (a disintegrin and metalloproteinase with thrombospondin motifs)-13, and complement factor H (CFH), a two-plex assay for tissue factor and thrombomodulin, and a four-plex assay for von Willebrand’s factor (vWF), c-reactive protein (CRP), Fibrinogen, and platelet factor (PF)-4 (Millipore). One single-plex assay for IFN-β, an eleven-plex assay for E-selectin, fractalkine, granzyme B, melanoma growth stimulation activity alpha (GRO-α), IL-29, L-selectin, MCP-2, MCP-3, sCD40L, TNF-R2, tissue plasminogen activator (tPA), and a six-plex assay for D-dimer, plasminogen activator inhibitor (PAI-1), platelet endothelial cell adhesion molecule (PECAM), P-selectin, sFas-ligand, TNF receptor (TNF-R1) were performed using a 2 hour incubation (Affymetrix). A two-plex assay for intercellular adhesion molecule (ICAM) and vascular cell adhesion molecule (VCAM) was run with an overnight incubation (Affymetrix). Data were collected on a Luminex 200 (Austin, TX) and all assay results were reported in pg/mL, ng/mL, or mg/dL. For 11 of the biomarkers evaluated (IFN-β, G-CSF, IL-5, IL-13, IL-17, GM-CSF, TNF-α, IL-2, IL-7, IL-4, and tissue factor) levels of the biomarker were near or below the limit of detection in all patient samples, so data are not presented graphically.

### Virus titer determination

RNA was isolated from gamma-irradiated whole blood using MagMax Total RNA Isolation Kit (Life Technologies, Grand Island, NY). qRT-PCR of RNA was conducted with established primer and probe sets for the RVFV L segment [23]. A standard curve, expressed as tissue culture infective dose 50 (TCID_50_)/mL, was generated from normal human whole blood spiked with a wild-type RVFV stock. The standard curve was used to convert raw Ct values to relative TCID_50_/mL.

### ELISA

Maxisorp plates (Nalgene-Nunc, Rochester, NY) were coated with RVFV lysate prepared from infected Vero-E6 cells as previously described [24], diluted 1:2000 in PBS, and allowed to adsorb overnight at 4°C. Plates were blocked in 5% milk in PBS with 0.1% Tween-20 (PBST) with 5% FBS for 1 hour at 37°C. Patient samples and controls were serially diluted in blocking buffer and incubated on plates for 2 hours at 37°C. Plates were washed three times in PBST then incubated for 1 hour at 37°C with anti-human IgG HRP or anti-human IgM HRP (Jackson ImmunoResearch Inc, West Grove, PA) diluted 1:5000 in blocking solution. Plates were washed three times in PBST before incubation in TMB substrate (KPL, Milford, MA) for 10 minutes. Reactions were stopped with TMB stop solution, and plates were read at 450nm. Data were analyzed using Excel (Microsoft Corp, Redmond, WA) and Prism (GraphPad Software Inc, La Jolla, CA).

### Statistical analysis

Descriptive analyses were performed using STATA 13.0 (StataCorp, College Station, TX) and Prism. Patient means were compared to those of the controls. P-values for all statistically significant comparisons are included in the supplementary table (S1 Table). Patient biomarkers were analyzed for correlation with RVFV viral load using Spearman’s rank correlation. Control biomarker values were compared to case values during two disease periods: days 7–19 and days 20–33. These time frames were chosen because virus was isolated in all cases until day 20. Wilcoxon rank sum was used to determine the significance of differences in control biomarker levels versus case biomarker levels. The False Discovery Rate technique was used to adjust for multiple comparisons with an overall alpha of 0.05 [25].

## Results

### Case 1

Case 1 was a middle aged male butcher who presented to a local hospital in Kabale District with a chief complaint of epistaxis. He had been well until a week prior to admission when he developed a high-grade fever associated with headache, neck pain, joint pain, and worsening fatigue. His admission labs were notable for an elevated white blood cell count of 16,100/μl, a decreased hemoglobin of 7.9 g/dl, and decreased platelets of 29,000/μl. Liver function tests revealed an elevated total bilirubin of 16.8 mg/dl and elevated transaminases. Creatinine and blood urea nitrogen were increased at 12.5 mg/dl and 240.5 mg/dl, respectively. Testing for malaria was negative. He was transferred to another hospital for transfusion and further management and recovered.

### Case 2

Case 2 is a teenage student who presented to a local hospital in Kabale with a five day history of fever, myalgia, joint pain, headache and diarrhea as well as three days of epistaxis. He had cared for goats and sheep at home. On admission he was noted to have epistaxis and scleral icterus. Laboratory evaluation revealed an elevated unconjugated hyperbilirubinemia of 9.72 mg/dl, elevated AST of 470 U/L, decreased total protein of 3.43 g/dl, elevated amylase of 386 U/L, decreased hemoglobin of 7.3 g/dl, and decreased platelets of 45,000/μL. Renal function was normal. He was treated empirically with broad spectrum antibiotics and required blood transfusions. His hospital course was complicated by seizures and altered mental status, but gradually improved.

### Case 3

Case 3 is a young adult builder who presented to a local hospital with fever, vomiting, abdominal pain, arthralgias, myalgias, and photosensitivity. He also had a history of epistaxis and hematemesis. Laboratory data were not available for this case.

### Viral loads and ELISA titers

**Fig 1:**
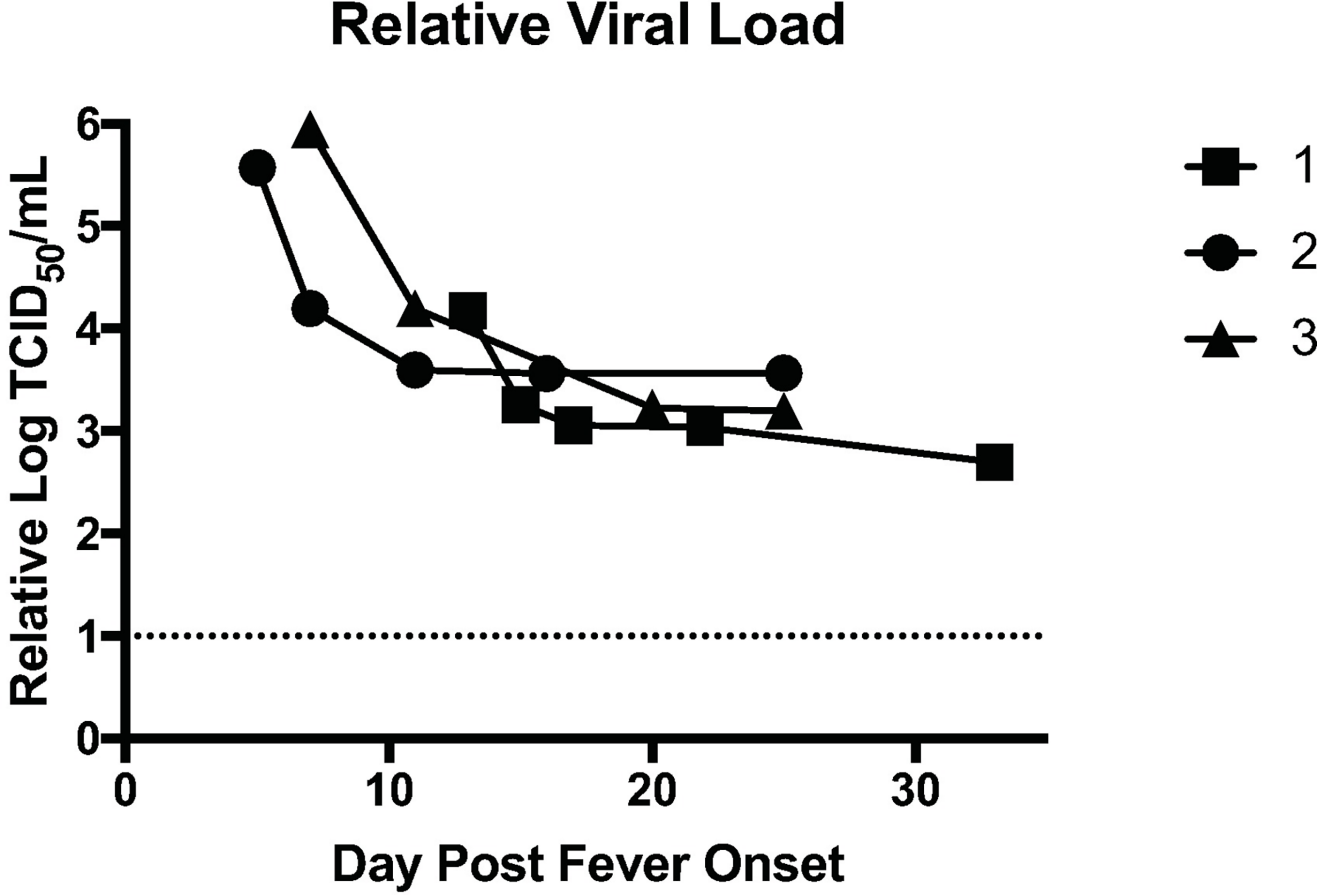
Kinetics of viral RNA load in the blood of patients. qRT-PCR data from each patient sample was converted to relative TCID_50_/mL by comparison with a standard curve generated from a virus stock of known titer. Each patient’s relative viral load was plotted as function of days post fever onset.

**Fig 2:**
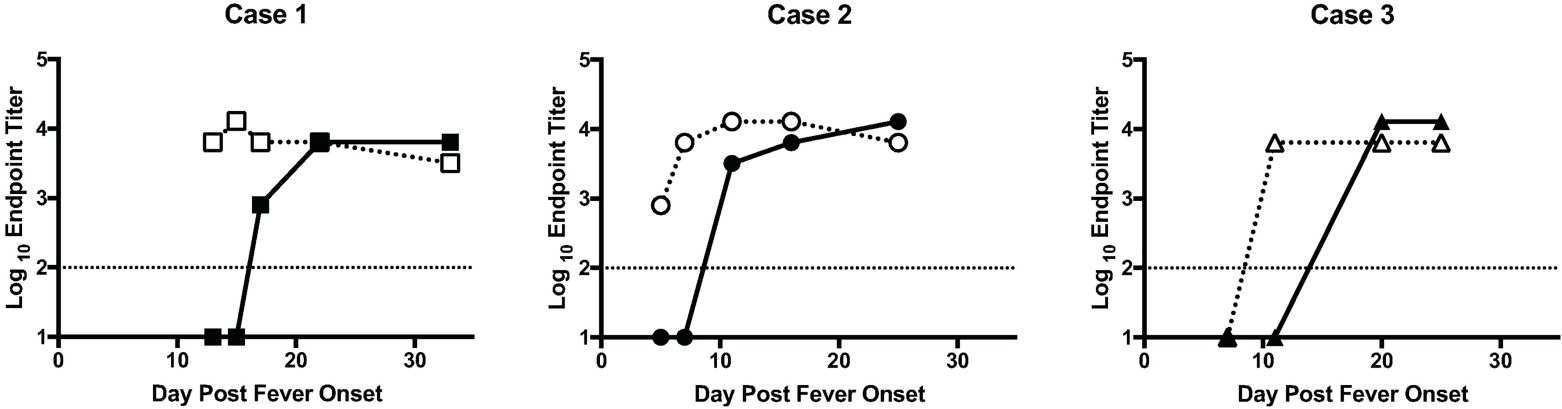
Kinetics of RVFV specific antibody responses in the blood of patients. ELISA endpoint titers are shown for each patient for both IgM (dotted line and open shape) and IgG (solid line and solid shape) as function of days post fever onset. The limit of detection of the assay is noted at 100, which is the lowest level at which the assay can accurately detect the presence of antibody.

The relative viral loads (log TCID_50_/mL) in each of the three cases decreased over time (Fig 1). All patients still had detectable viral RNA after disease resolution, which likely represents the sensitivity of the assay rather than persistence of infectious virus since viral isolation attempts before day 20 post fever onset were successful and isolation attempts at time points 20 and more days post fever onset were unsuccessful. RVFV IgM was detectable on first date of blood sampling for Cases 1 and 2, at day 13 for Case 1, day 5 for Case 2, but not until the second blood sample for Case 3, on day 11 (Fig 2). RVFV IgG was undetectable in all three cases prior to 10 days post fever onset but was detectable at day 17 for Case 1, day 11 for Case 2, and day 20 for Case 3 (Fig 2).

### Correlation of biomarkers with viral load

Correlation of biomarkers with viral load was assessed. Only those that were significant after adjusting for multiple comparisons are presented in Table 1. sICAM-1, CRP, IP-10, eotaxin, tPA, D-dimer, granzyme B, and MCP-2 were all significantly positively correlated with viral load. P-selectin, ADAMTS13, CFH, and Fibrinogen were all significantly negatively correlated with viral load. Of note, three of these factors (CRP, P-selectin and CFH) did not vary significantly outside the range of normal.

**Table 1:** Correlation of biomarkers with viral load.

Biomarker	Correlation Coefficient	p-value
sICAM-1	0.9165	<0.0001
CRP	0.8066	0.0005
IP-10	0.789	0.0008
Eotaxin	0.7415	0.0024
tPA	0.6835	0.007
D-dimer	0.6791	0.0076
Granzyme B	0.6696	0.0088
MCP-2	0.6615	0.01
P-selectin	-0.6484	0.0121
ADAMTS13	-0.6527	0.0114
CFH	-0.6923	0.0061
Fibrinogen	-0.8286	0.0003

sICAM (soluble intracellular adhesion molecule); CRP (c-reactive protein); IP (interferon-γ induced protein); tPA (tissue plasminogen activator); MCP (monocyte chemoattractant protein); ADAMTS (a disintegrin and metalloproteinase with thrombospondin motifs); CFH (complement factor H).

### Biomarkers of coagulopathy

**Fig 3:**
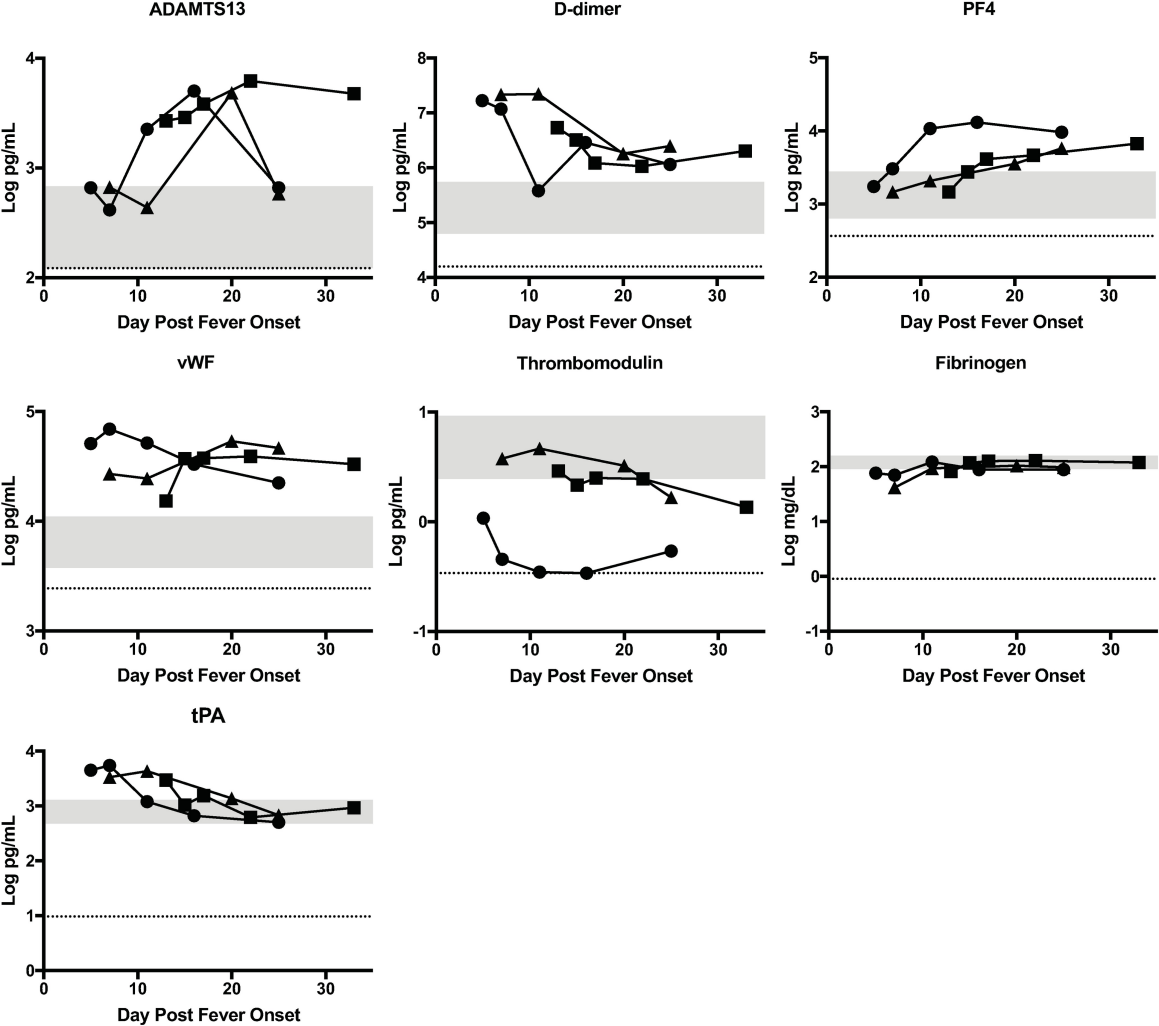
Statistically significant biomarkers of coagulopathy in RVF cases. The concentration of each biomarker in each patient is plotted as a function of day post fever onset; Case 1 (square), Case 2 (circle), Case 3 (triangle). The grey area represents the range of the biomarker concentration that was detected in 8 normal healthy individuals. The dotted line is the limit of detection of the assay, which is the lowest level at which the assay can accurately detect the presence of the biomarker.

Hemostatic derangement has been observed in human RVF cases, and was noted in all three of these patients. Although some markers of coagulopathy have been studied in rhesus monkeys [26], there is limited data regarding human markers of coagulopathy during RVFV infection. For this reason, case biomarkers of coagulopathy were compared to control levels during two disease periods: days 7–19 (virus isolation positive) and days 20–33 (virus isolation negative). Only data in which patient values were significantly outside the normal range are shown in the main figures. Any data obtained but not included in the main figures are presented in the Supplementary material for completeness (S1 Fig). Levels of ADAMTS13, D-dimer, PF4, and vWF were significantly elevated in cases compared to controls during both disease periods (Fig 3). Thrombomodulin was significantly lower in cases compared to controls in both disease periods, but this data appears to be skewed by Case 2 with much lower levels than the others. Fibrinogen levels in cases were significantly lower than control levels early in the disease course, however mean fibrinogen levels increased in cases and were no longer significantly different from control levels after day 20. In contrast, tPA levels in cases were significantly elevated early during the course of illness and decreased such that mean case levels were within the normal range later in the disease course.

### Endothelial markers

**Fig 4:**
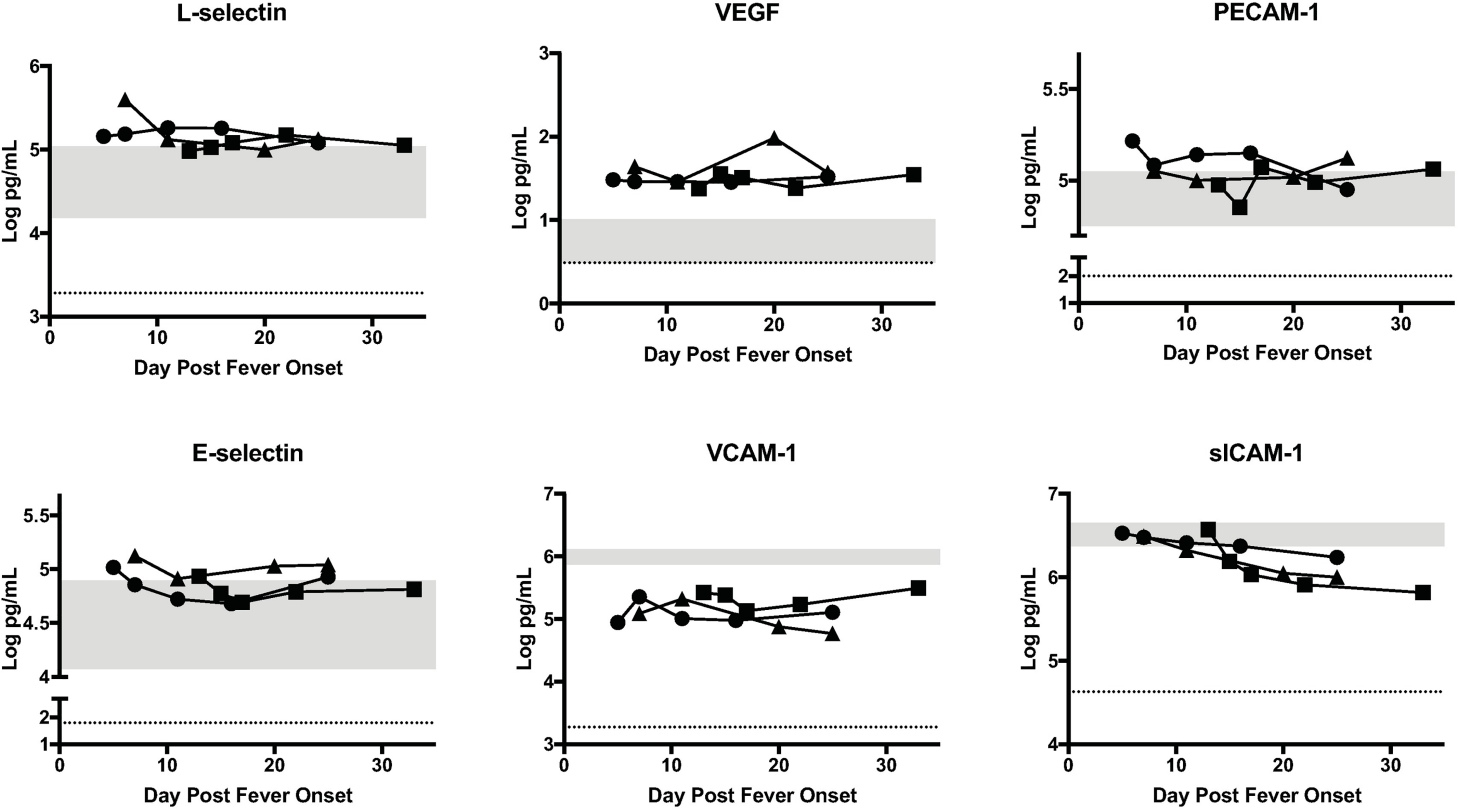
Statistically significant biomarkers of endothelial function in RVF cases. The concentration of each biomarker in each patient is plotted as function of day post fever onset; Case 1 (square), Case 2 (circle), Case 3 (triangle). The grey area represents the range of the biomarker concentration that was detected in 8 normal healthy individuals. The dotted line is the limit of detection of the assay, which is the lowest level at which the assay can accurately detect the presence of the biomarker.

Endothelial expression of cell surface molecules can modify vascular stability, coagulation, and leukocyte recruitment [27]. Upregulation of these molecules can be associated with infection and inflammation. Given that microscopic changes in the endothelium have been noted in monkeys infected with RVFV [28], case endothelial marker levels were compared to control endothelial marker levels in the two disease periods. Only data in which patient values were significantly outside the normal range are shown in the main figures. Any data obtained but not included in the main figures is presented in Supplementary material for completeness. Variations in endothelial marker levels were seen during both phases of illness among cases (Fig 4). L-selectin and VEGF levels were significantly higher in cases than controls throughout the illness. However, PECAM-1 was only significantly elevated early in the disease in cases compared to controls. In contrast, case E-selectin levels were normal early in the illness course but increased over time and were significantly elevated in cases late in the course of illness. Several endothelial markers were decreased among cases compared to controls. Mean VCAM levels were significantly lower in cases compared to controls throughout the course of illness. Soluble ICAM-1 levels were within the normal range early in the course of illness but decreased significantly late in the course of illness among cases compared to controls.

### Chemokines and cytokines

Chemokines and cytokines play a vital role in recruitment, activation and maturation of the immune system; for this reason, we compared case levels of a series of cytokines and chemokines to control levels. Only data in which patient values were significantly different from controls are shown in the main figures. Any data obtained but not included in the main figures are presented in the Supplementary material for completeness.

**Fig 5:**
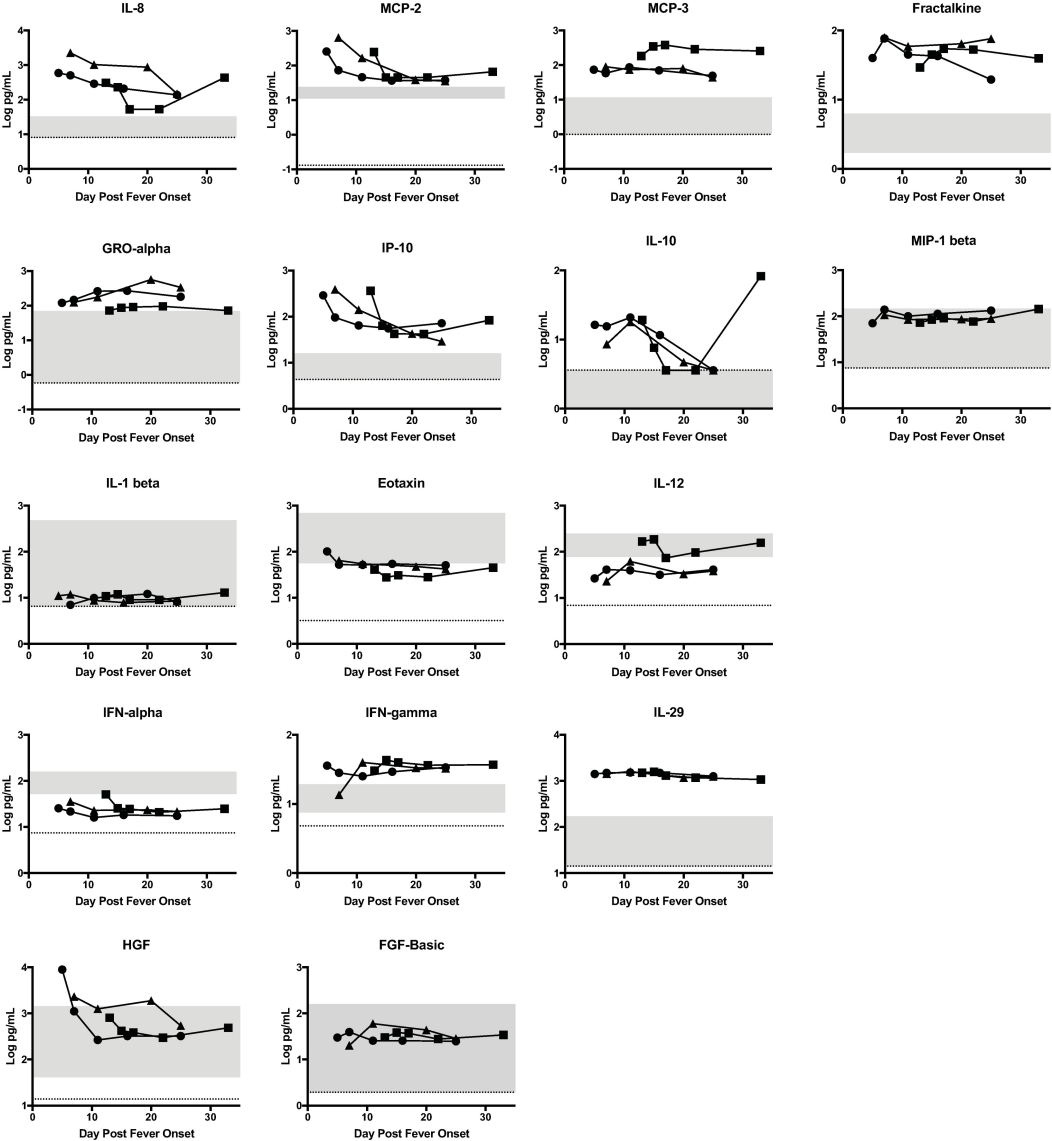
Statistically significant cytokines, chemokines and growth factor levels of in RVFV cases. The concentration of each biomarker in each patient is plotted as function of day post fever onset; Case 1 (square), Case 2 (circle), Case 3 (triangle). The grey area represents the range of the biomarker concentration that was detected in 8 normal healthy individuals. The dotted line is the limit of detection of the assay, which is the lowest level at which the assay can accurately detect the presence of the biomarker. For some of these biomarkers, the shaded area is below the dotted line because the control levels were not detectable above the limit of detection suggesting that these biomarkers are not detectable in healthy individuals.

Case levels of IL-8, MCP-2, MCP-3, fractalkine, GRO-α, and IP-10 were significantly elevated when compared to control levels throughout the course of illness (Fig 5). IL-10 and MIP-1β levels in cases were significantly elevated compared to control levels early during the illness course. However MIP-1β levels were within the normal range and did not exhibit marked variation during the disease course. Certain chemokines and cytokines were significantly lower in cases compared to controls. Case IL-1β and eotaxin levels were lower throughout the disease course. IL-12 levels in cases were significantly lower early in the course of illness only.

RVFV infection *in vitro* and in animal models has been associated with decreased expression of type 1 interferons, secondary to the interferon antagonism activity of the RVFV NSs protein [8]. Consistent with this, IFN-α was lower in cases compared to controls. IFN- γ and IL-29, a type III interferon, were noted to be higher in cases compared to controls but did not fluctuate significantly during the clinical course.

Our assays also examined several different types of growth factors. Mean HGF levels in cases were significantly elevated early during the course of illness only. Mean case levels of FGF-basic were significantly elevated compared to mean control levels throughout the illness course; however, they were still within the range of control values.

### Other biomarkers

**Fig 6:**
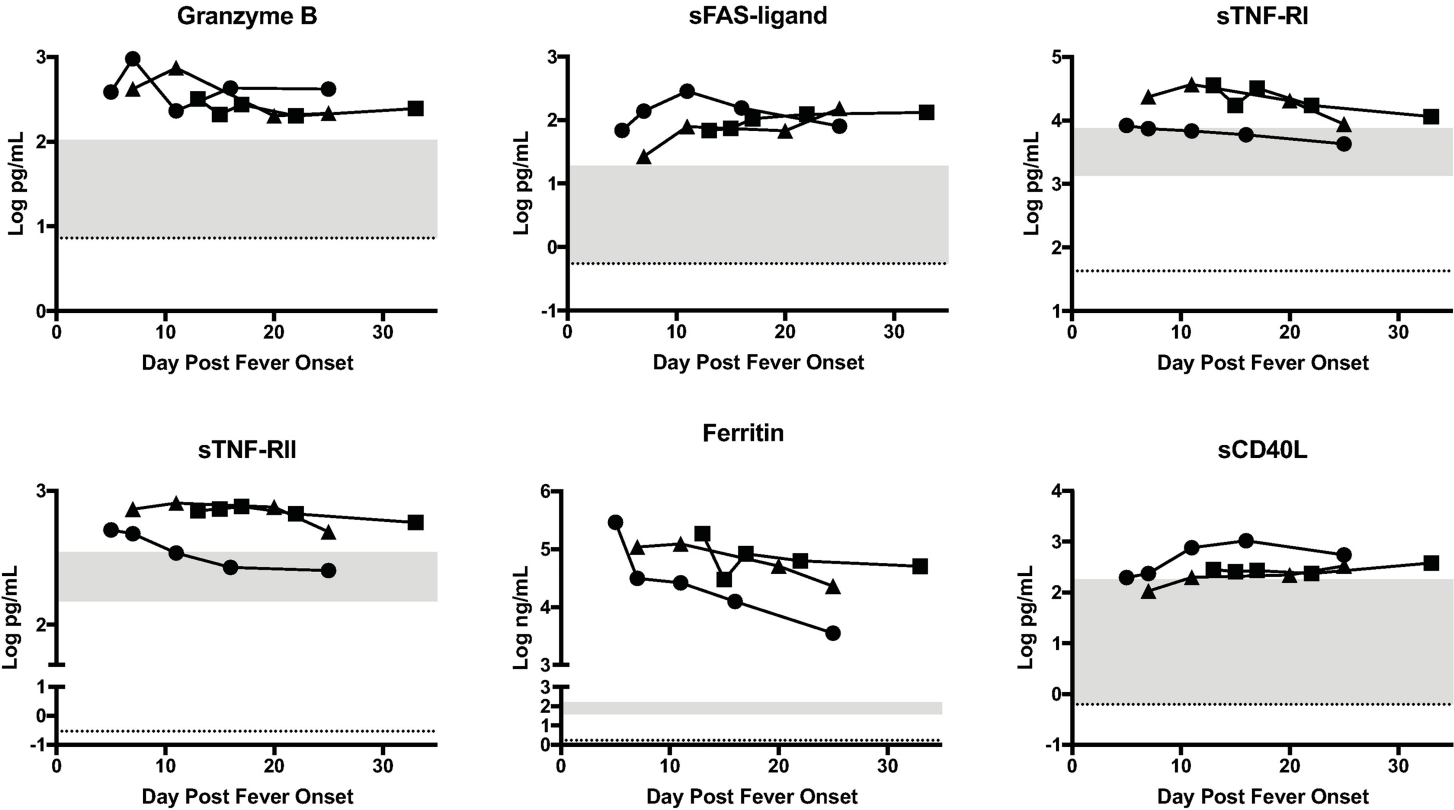
Statistically significant biomarkers of apoptosis and inflammation in RVF cases. The concentration of each biomarker in each patient is plotted as function of day post fever onset; Case 1 (square), Case 2 (circle), Case 3 (triangle). The grey area represents the range of the biomarker concentration that was detected in 8 normal healthy individuals. The dotted line is the limit of detection of the assay, which is the lowest level that the assay can accurately detect the presence of the biomarker.

Granzyme B, a protease found in cytotoxic lymphocytes and natural killer cells that mediates apoptosis, and three other mediators of apoptosis, sFAS-ligand, sTNF-RI and sTNF-RII were found to be significantly elevated in cases compared to controls (Fig 6).

We also measured non-specific markers of inflammation including C reactive protein (CRP) and ferritin (Supplementary material and Fig 6). Although case CRP levels were within the normal range and were not significantly elevated compared to mean control CRP levels, they did tend to be elevated early in the disease and decreased over time. Ferritin levels were significantly elevated in cases throughout the illness. sCD40L, which is mostly derived from activated platelets, was elevated in all patients throughout the course of infection.

## Discussion

In this study, we evaluated a series of biomarkers in three patients to examine several aspects of human pathophysiology during primary RVFV infection. All three cases had severe disease with hemorrhagic manifestations, required hospitalization, and all recovered from their illness. Although our study only included three cases, our findings illuminate the interaction of RVFV with the immune system, endothelium, and coagulation pathways.

The pro-inflammatory cytokines/chemokines IL-8, IL-10, IP-10, MCP-2, MCP-3, fractalkine, and GRO-α were all significantly elevated in cases compared to controls. MCP-2 and IP-10 were elevated in all three cases and correlated with viral load, suggesting that as viremia resolved, the inflammatory response normalized. IL-8, MCP-1, MCP-2, TNF-R1, IP-10, and sFAS-ligand have been previously studied in Ebola, dengue, and Lassa patients and have shown some correlation with disease severity [11, 13, 17, 29]. A previous study found IL-8, IL-10, IP-10, CXCL9 and MCP-1 to be increased significantly in fatal RVF cases [18] however other cytokines/chemokines have not been studied extensively in RVF. Although we were not able to correlate these biomarkers with disease severity given the small numbers of patients, we observed elevated levels of these same pro-inflammatory molecules during the acute illness and trends of these biomarkers show normalization of levels as patients recovered.

IL-1β, IL-12, and eotaxin, were lower in cases than in controls. Of these only eotaxin was correlated with viral load. Eotaxin is a pro-inflammatory cytokine that mediates chemotaxis of eosinophils, basophils, mast cells, and Th2 lymphocytes during infection and has been noted to be lower in some patients with dengue infection when compared to controls, particularly patients with significant vascular leakage [30]. Further research using larger numbers of patients should be conducted to understand if RVF disease severity or clinical manifestations correlate with expression of these cytokines.

Data from the literature suggests that RVFV infection is associated with decreased expression of interferons, secondary to the activity of the RVFV NSs protein [8]. IFN-α and IFN-β are an important part of the innate immune response to viral infection. Our study found that IFN-α was low throughout the course of illness and IFN-β was undetectable; it is tempting to speculate that this might be secondary to viral suppression via the NSs protein. Interestingly, IL-29, a type III interferon, was elevated in our cases. Type III interferons primarily target mucosal epithelial cells and the liver; polymorphisms in certain Type III interferons have been associated with differential HCV clearance [31]. Our cases demonstrated elevated IL-29 and IFN-γ throughout the course of illness and no significant changes in levels were observed over time, therefore elevated IL-29 and IFN-γ levels observed in our study may be due to persistent immune activation or could simply be due to underlying differences in the Ugandan versus North American populations, rather than related to disease course.

We found that levels of endothelial markers including P-selectin and sICAM-1 correlated with viral load. sICAM-1 levels decreased through the clinical course suggesting that endothelial activation and damage improved as viral infection resolved. In contrast, P-selectin levels increased as viral load decreased. The increase could contribute to resolution of coagulopathy in these RVF survivors, since P-selectin is involved in anchoring vWF to the endothelial cell surface thereby aiding in cleavage of vWF, which plays a role in clot formation [32]. Endothelial markers have not been studied previously in RVFV infections, but have been studied in Ebola [11, 13] and dengue [15, 33, 34] infections. Previous studies have demonstrated that elevated sICAM levels in dengue [15] and Ebola cases [11, 13] were associated with more severe disease. We were unable to correlate levels of endothelial markers with clinical severity; however our findings suggest that endothelial dysfunction may play a role in RVF pathogenesis.

Given the fact that all three patients had hemorrhagic symptoms, it is not surprising that cases had higher levels of biomarkers of coagulopathy than controls. D-dimer and tPA were all elevated in cases compared to controls on presentation and were significantly correlated with viral load. Our observation of elevated d-dimer in cases is similar to findings in Ebola patients and dengue patients[12, 35] and is indicative of ongoing fibrinolysis. Elevated levels of tPA are also consistent with the increased fibrinolysis. Laboratory evidence of decreased clotting and increased fibrinolysis on presentation is consistent with the bleeding that was initially observed in all three patients. tPA and D-dimer levels decreased over time and correlated with viral load decline, suggesting that clinical improvement and viremia resolution may correlate with decreased fibrinolysis.

Elevated levels of vWF are less informative since the assay does not distinguish between the pro-coagulant multimers of vWF and the cleaved forms. It is interesting that levels of ADAMTS13, which is a protease that cleaves vWF, increased with disease resolution in all three patients and then declined to normal in two of the patients. This could be an indicator of correction of the coagulopathy that was noted during acute disease. In support of this hypothesis, in a study of pediatric patients with severe dengue, ADAMTS13 activity was noted to be lower than normal, presumably secondary to consumption [14]. Deficiencies in ADAMTS13 levels or function can result in clinical manifestations of bleeding paradoxically via formation of microthrombii. As mentioned previously, P-selectin also negatively correlated with viral load (i.e. levels increased as disease resolved). P-selectin is produced by activated platelets and increases in this protein could, similar to increases in ADAMTS13, indicate a resolution of the coagulopathy that was noted clinically in the patients. This analysis suggests that hemorrhagic symptoms in RVF are due to complex interactions between the virus and both coagulation and fibrinolytic pathways.

Granzyme B, a marker of cytotoxic T cell function, was also elevated in all patients and correlated with viral load decline. This finding is interesting because in patients with Ebola virus disease, both disease severity and viremia were correlated with granzyme B levels [11]. This could simply be a reflection of higher viral loads providing more antigen stimulus to T cells and thus more cytotoxic T cell activity and may be an indirect marker of disease severity. However, there are no data on the role of T cells during human RVFV infection, indicating a gap in our knowledge of RVFV pathophysiology that clearly warrants additional study.

There are several limitations to our study. We had limited samples from only three patients, and we did not have complete clinical and laboratory information from all of the patients. Additionally, the healthy controls were from the United States and may have different baseline laboratory values of the biomarkers tested due to genetic or environmental differences than a healthy Ugandan. Given the limited number of patients and the fact that all three were ill enough to require hospitalization, we were unable to draw conclusions between biomarkers and severity of disease. Although our study had few patients, we were able to demonstrate similar trends in these cases in order to gain insight into the pathogenesis of symptomatic RVFV infection.

We found that RVFV infection appears to have a complex and at times contradictory impact on endothelial markers, immune response, and coagulation. Further studies should be done to better characterize these biological pathways during RVFV infection. These types of studies have promise in determining if these biomarkers are associated with clinical outcome in patients with RVFV infection and whether they may serve as prognostic indicators of disease or possibly as clues that could guide the use of host-directed therapeutics.

## Supporting information

S1 Fig Non-statistically significant biomarkers in RVF cases. The concentration of each biomarker in each patient is plotted as function of day post fever onset; Case 1 (square), Case 2 (circle), Case 3 (triangle). The grey area represents the range of the biomarker concentration that was detected in 8 normal healthy individuals. The dotted line is the limit of detection of the assay.
(PDF)

S1 Table Summary of p-values for all statistically significant analytes during both time periods in RVF patients. Mean analyte value from patients was compared to mean analyte value from controls. Any value in italics did not meet criteria for significance for the indicated disease period.
(PDF)
