# Supplementary figures and images for: Rift valley fever viral load correlates with the human inflammatory response and coagulation pathway abnormalities in humans with hemorrhagic manifestations
Source: PLoS Negl Trop Dis. 2018 May 4;12(5):e0006460. doi: 10.1371/journal.pntd.0006460 (PMC5955566; doi:10.1371/journal.pntd.0006460)

# Supplementary Figure

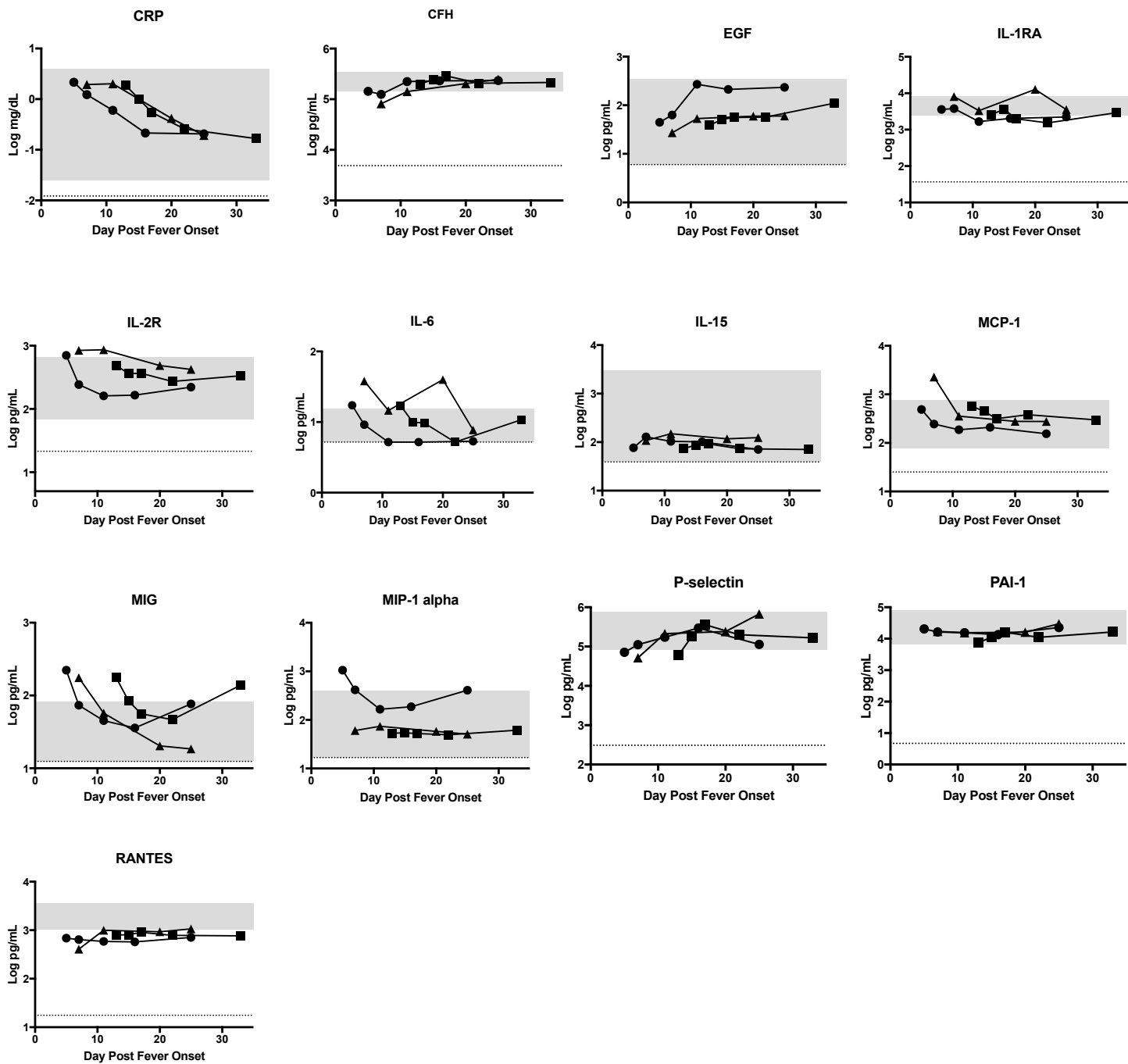

Supplement: S1 Fig — The concentration of each biomarker in each patient is plotted as function of day post fever onset; Case 1 (square), Case 2 (circle), Case 3 (triangle). The grey area represents the range of the biomarker concentration that was detected in 8 normal healthy individuals. The dotted line is the limit of detection of the assay. (PDF) [file pntd.0006460.s001.pdf]
