# Supplementary material for: Rift valley fever viral load correlates with the human inflammatory response and coagulation pathway abnormalities in humans with hemorrhagic manifestations
Source: PLoS Negl Trop Dis. 2018 May 4;12(5):e0006460. doi: 10.1371/journal.pntd.0006460 (PMC5955566; doi:10.1371/journal.pntd.0006460)
Supplement: S1 Table — Mean analyte value from patients was compared to mean analyte value from controls. Any value in italics did not meet criteria for significance for the indicated disease period. (PDF) [file pntd.0006460.s002.pdf]

Analyte	p-value period 1	p-value period 2
ADAMTS13	0.0269	0.0008
D-Dimer	0.0008	0.0034
Eotaxin	0.0021	0.0034
E-selectin	*0.0543*	0.0192
Ferritin	0.0005	0.0034
FGF-Basic	0.0071	0.0170
Fibrinogen	0.0161	*0.1877*
Fractalkine	0.0008	0.0045
Granzyme-B	0.0005	0.0026
GRO-α	0.0005	0.0034
HGF	0.0206	*0.0773*
ICAM	*0.6304*	0.0084
IFN-α	0.0052	0.0192
IFN-γ	0.0019	0.0029
IL-8	0.0003	0.0015
IL-10	0.0005	0.0034
IL-12	0.0124	*0.0404*
IL-1β	0.0005	0.0029
IL-29	0.0005	0.0033
IP-10	0.0005	0.0034
L-selectin	0.0005	0.0034
MCP-2	0.0005	0.0034
MCP-3	0.0005	0.0029
MIP-1β	0.0163	*0.0388*
PECAM-1	0.0161	*0.0790*
PF-4	0.0209	0.0034
sCD40L	0.0011	0.0034
sFAS-L	0.0005	0.0033
Thrombomodulin	0.0007	0.0130
TNF-R1	0.0015	0.0190
TNF-RII	0.0021	0.0192
tPA	0.0053	*0.7697*
VCAM	0.0005	0.0034
VEGF	0.0005	0.0034
vWF	0.0005	0.0034

Any value in italics did not meet criteria for significance for the indicated disease period.
